# Evaluation of Measurement Uncertainty in Clinical Chemistry and its Comparison With Analytical Performance Specifications

**DOI:** 10.7759/cureus.79043

**Published:** 2025-02-15

**Authors:** Nishtha Wadhwa, Kiran Bhat, Mansi Kalsi, Tushita Sadhu

**Affiliations:** 1 Biochemistry, Himalayan Institute of Medical Sciences, Dehradun, IND; 2 Biochemistry, Shri Guru Ram Rai Institute of Medical and Health Science, Dehradun, IND

**Keywords:** analytical performance specifications, measurement uncertainty, metrological traceability, quality control, uncertainty

## Abstract

Introduction

To evaluate the variability in values reported by a clinical laboratory, it is important to assess the measurement uncertainty (MU). Furthermore, verifying that the estimated MU aligns with relevant analytical performance specifications (APS) is essential. Previous research has indicated variability in how laboratories estimate and apply MU, with some failing to meet APS, potentially affecting clinical decision-making. Additionally, factors such as differences in instrumentation, reagent quality, and calibration protocols may contribute to regional variations in MU, emphasizing the need for a systematic evaluation. This study aimed to determine the MU of 31 measurands and compare it with the APS available in the literature to assess whether our measuring systems can meet these APS.

Methods

The study was conducted in the Clinical Biochemistry Laboratory of the Himalayan Institute of Medical Sciences, Dehradun, India. The measuring systems in our laboratory are Beckman Coulter DxC 700 AU (Beckman Coulter, Inc., Brea, California, United States), Beckman Coulter UniCel DxI 800 (Beckman Coulter, Inc.,), and bioMérieux VIDAS (bioMérieux SA, Marcy-l'Étoile, France). The ‘top-down’ approach which uses internal quality control data and calibrator information was used for the estimation of MU. The formula used was: *u*_result _=√(*u*_cal_^2^ + *u*_Rw_^2^+ *u*_bias_^2^). Objectively derived APS available in the literature were utilized to determine the allowable MU.

Results

A total of 24 measurands exhibited uncertainty estimates below the minimum APS limits. However, serum lactate dehydrogenase (LD), creatine kinase (CK), alkaline phosphatase (ALP), sodium, chloride, glucose, and ferritin showed MU higher than the minimum APS. The calibrator uncertainties (u_cal_) for ALP (4.7), LD (5.45), CK (11.06), and Ferritin (23.2) were significantly high, contributing to their elevated MU estimates. Additionally, the minimum APS for serum chloride (0.74) and sodium (0.40) were particularly stringent and could not be met.

Conclusion

The evaluation of MU provides objective insights into the quality of measurement systems and, its comparison, against set APS, supports the applicability of laboratory results in clinical decision-making. Failing to meet APS can lead to misdiagnosis, treatment errors, and patient safety risks. It may also hinder ISO 15189 accreditation and increase retesting costs.

## Introduction

Laboratory results play a crucial role in clinical decision-making by providing objective, evidence-based data that guide diagnosis, treatment selection, and disease monitoring. [1,2]. Therefore, these results must be reliable (accurate) and comparable with those obtained at different times and locations (traceable) [3,4].

Hence, the main objective of laboratory medicine is to prevent measurement variability from overshadowing the clinical information provided by test results [5,6]. To ensure the safety and usefulness of measurement results in clinical practice, and to enable meaningful comparisons with clinical decision limits and previous results for the same individual, clinical laboratories must estimate the variability in the values reported by their measurement systems [7]. Estimating measurement uncertainty (MU) in laboratory results is crucial for obtaining accreditation under the ISO 15189:2022 standard [5,8]. This process not only aligns with international quality requirements but also enhances confidence in the validity of measurement results [9,10].

MU is defined as the “non-negative parameter characterizing the dispersion of the quantity values being attributed to a measurand based on the information used.” This definition suggests that when interpreting a measurement result accompanied by an uncertainty value, the "true value" lies within the given uncertainty range, with a specified probability level [11,12].

Various methodologies exist for calculating MU, including the Nordtest approach and the 'Top-Down' approach outlined by the ISO Technical Specification 20914:2019. Additionally, the Unity Real Time software by Bio-Rad facilitates the calculation of standard and combined uncertainties. Among these methods, the 'Top-Down' approach recommended by ISO is generally regarded as the most suitable for application in clinical laboratories. This method incorporates all sources of uncertainty within the selected traceability chain. It uses the commercial calibrator information along with the internal quality control (IQC) data which identifies the random component of uncertainty [7,9]. It also stresses that the estimated MU must be appropriate for medical decision-making. Therefore, the estimated MU should be compared with the allowable MU, defined by objectively determined analytical performance specifications (APS) [7].

APS are essential in clinical laboratories to ensure test results are accurate, reliable, and clinically relevant. They provide objective criteria for evaluating MU, ensuring results meet medical decision-making needs. APS standardize test quality across laboratories, support regulatory compliance (e.g., ISO 15189), and enhance patient safety by minimizing errors. By defining allowable MU, APS help manage risk, ensuring laboratory tests contribute effectively to diagnosis and treatment. Three models for establishing APS were proposed at the strategic conference held in Milan by the European Federation of Clinical Chemistry and Laboratory Medicine (EFLM) in 2014 [13]. Model 1 emphasizes how analytical performance directly influences clinical outcomes. Model 2 is derived from the components of biological variation (BV) of the measurands. Model 3 relies on the state of the art in measurement, representing the highest level of analytically achievable performance. While Model 1 is designed for measurands crucial for diagnosing and monitoring specific diseases, Model 2 is suitable for measurands under tight metabolic control and Model 3 applies to measurands that do not fit into the first two models' categories [5,13].

The EFLM Biological Variation Database provides the maximum allowable MU for various measurands based on biological variation data [14]. In their publications in Clinical Chemistry and Laboratory Medicine, Braga et al. categorized several analytes according to the Milan models and established APS for MU by thoroughly reviewing existing literature and carefully selecting relevant data [5,6]. 

ISO 15189:2022 clause 7.3.4 on the evaluation of MU requires laboratories to document the comparison of evaluated MU with performance specifications [8]. Meeting APS for MU ensures that laboratory results are reliable and clinically applicable. As the significance of standardization and traceability of test results grows, the consideration of MU is poised to emerge as a crucial aspect of laboratory quality management [7].

Previous research has indicated variability in how laboratories estimate and apply MU, with some failing to meet APS, potentially affecting clinical decision-making [5,6]. Additionally, factors such as differences in instrumentation, reagent quality, and calibration protocols may contribute to regional variations in MU, emphasizing the need for a systematic evaluation.

In this study, we evaluated the MU for 31 chemistry and immunoassay measurands and compared them with the defined APS for MU. By comparing MU across multiple measurands, this study aims to identify gaps in current practices and highlight areas for improvement in laboratory quality management.

## Materials and methods

This study was conducted in the Clinical Biochemistry Laboratory of the Himalayan Institute of Medical Sciences, Dehradun, India, following approval from the Institutional Ethics Committee (Ethical Clearance No. HIMS/RC/2024/77). The measuring systems in the laboratory were Beckman Coulter DxC 700 AU (Beckman Coulter, Inc., Brea, California, United States), Beckman Coulter UniCel DxI 800 (Beckman Coulter, Inc.), and bioMérieux VIDAS (bioMérieux SA, Marcy-l'Étoile, France). The laboratory used third-party internal quality control (IQC) materials from Bio-Rad Laboratories, Inc. (Hercules, California, United States). A total of 31 commonly used measurands were selected for which MU was estimated and compared with APS available in the literature.

Evaluation of MU

The ‘top-down’ approach which uses commercial calibrator information and IQC data was used for the estimation of MU as recommended by ISO 20914:2019 [7]. The formula used was:

u_result_ =√(u_cal_^2^ + u_Rw _^2^+ u_bias_^2^)

Where,

u_cal_ refers to the uncertainty associated with the value that is assigned to an end-use calibrator. This information was obtained from the manufacturer upon request.

u_Rw_ represents the estimated standard uncertainty for a specific measuring system within the same laboratory over an extended period. This estimate accounts for routine changes in measuring conditions such as reagent lot changes, calibrators, instrument maintenance, operators, and environmental conditions. The uRw values were calculated from the standard deviation (SD) of daily IQC data for each measurand across two (chemistry) or three (immunoassay) levels of quality control (QC) from January to December 2023.

u_bias_ represents the uncertainty associated with any bias correction implemented by the laboratory. In our laboratory, external quality assurance performance and peer review on Unity Real Time software (Bio-Rad Laboratories, Inc.) indicated that none of the measurands exhibited medically significant bias. Therefore, no bias corrections were performed.

Source of APS

In this study, the allowable MU for 21 measurands was determined using the APS derived by Braga et al., based on the models established by the 2014 consensus conference of the EFLM [5,6]. For the remaining 10 measurands, the allowable MU was sourced from the EFLM Biological Variation Database [14].

## Results

APS for serum total cholesterol and plasma glucose were outcome-based (Milan model 1). For all other measurands, APS were derived from Biological Variation data (Milan model 2). For some measurands belonging to Milan Model 1, outcome-based data were not available in the literature, and hence Biological Variation data were used for allowable MU. The list of measurands, source of APS, and desirable and minimum APS are shown in Table 1.

**Table 1:** Analytical performance specifications (APS) for the evaluated measurands. EFLM: European Federation of Clinical Chemistry and Laboratory Medicine

MEASURAND	SOURCE OF APS	DESIRABLE APS (%)	MINIMUM APS (%)
Serum Alkaline Phosphatase	Biological variation	2.65	3.98
Serum Aspartate Aminotransferase	Biological variation	4.75	7.13
Serum Total Cholesterol	Outcome-Based	3	7
Serum High-Density Lipoprotein Cholesterol	Biological variation	2.84	4.26
Serum Triglycerides	Biological variation	9.9	14.9
Serum Albumin	Biological variation	1.25	1.88
Serum Total Proteins	Biological variation	1.3	1.95
Serum Urate	Biological variation	4.16	6.24
Plasma Glucose	Outcome-Based	2	3
Serum Creatinine	Biological variation	2.2	3.3
Serum Urea Nitrogen	Biological variation	7.05	10.6
Serum Total Bilirubin	Biological variation	10.5	15.7
Serum Total Calcium	Biological variation	0.91	1.36
Serum Alanine Aminotransferase	Biological variation	4.65	6.98
Serum Sodium	Biological variation	0.27	0.40
Serum Potassium	Biological variation	1.96	2.94
Serum Chloride	Biological variation	0.49	0.74
Serum Lactate Dehydrogenase	Biological variation	2.6	3.9
Serum Creatine Kinase	Biological variation	7.25	10.9
Serum magnesium	Biological variation	1.44	2.16
Serum Gamma Glutamyltransferase	Biological variation	4.45	6.68
Serum Phosphorus	EFLM Biological Variation Database	7.7	11.6
Serum Triiodothyronine, Free	EFLM Biological Variation Database	5.1	7.6
Serum Thyroxine, Free	EFLM Biological Variation Database	4.8	7.2
Serum Thyroid Stimulating Hormone	EFLM Biological Variation Database	17.9	26.8
Serum Luteinising Hormone	EFLM Biological Variation Database	25.2	37.8
Serum Follicle Stimulating Hormone	EFLM Biological Variation Database	9.5	14.3
Serum Prolactin	EFLM Biological Variation Database	45	67.5
Serum Ferritin	EFLM Biological Variation Database	12.9	19.4
Serum Total Prostate Specific Antigen	EFLM Biological Variation Database	6.8	10.2
Serum Cancer Antigen 125	EFLM Biological Variation Database	8.7	13

MU for all the measurands was calculated at all levels of IQC and compared with the allowable MU data (APS). Among 31 measurands evaluated, 24 achieved MU below the minimum APS limits. Among these, 23 had a MU below the desirable APS. The MU for serum total cholesterol (4.27 for L1 and 3.33 for L2) was below minimum APS (7) but above desirable APS (3). The method of measurement, calibrator traceability, u_cal_, u_Rw_, and u_result_ of the three measuring systems are presented in Tables 2-4.

**Table 2:** Characteristics of measuring systems and measurement uncertainty contributions for measurands evaluated in Beckman Coulter DxC 700 AU* ^a^ Values higher than the minimum APS are in bold; *Beckman Coulter, Inc., Brea, California, United States QC: quality control; U_Rw_: measurement uncertainty for long-term imprecision; U_cal_: measurement uncertainty of the value assigned to an end-use calibrator; U_result_: measurement uncertainty associated with laboratory results; IFCC: International Federation of Clinical Chemistry and Laboratory Medicine; IDMS: isotope dilution mass spectrometry; NIST:  National Institute of Standards and Technology; SRM: Standard Reference Material; CRM: Certified Reference Material: DPD: 2,4-dichlorophenyldiazonium: ISE: ion-selective electrode; PNPP: p-nitrophenyl phosphate; AMP: 2-amino-2-methyl-1-propanol; UV: ultraviolet; NAC: N-acetyl cysteine

MEASURAND	METHOD	CALIBRATOR TRACEABILITY	QC LEVEL	U_Rw_	U_cal_	U_result_^a^
Serum Alkaline Phosphatase	PNPP, AMP Buffer	IFCC reference measurement procedure	1	2.48	4.7	5.31
			2	9.86	4.7	10.92
Serum Aspartate Aminotransferase	UV without P5P	IFCC reference measurement procedure	1	1.19	1.51	1.92
			2	4.25	1.51	4.51
Serum Total Cholesterol	Cholesterol oxidase, esterase, peroxidase	NIST SRM 909b L1	1	2.95	3.09	4.27
			2	1.24	3.09	3.33
Serum High Density Lipoprotein Cholesterol	Direct measure, immunoinhibition	CDC reference method	1	1.11	1.3	1.71
			2	0.48	1.3	1.39
Serum Triglycerides	Enzymatic, endpoint	IDMS reference method	1	2.48	4.42	5.07
			2	1.75	4.42	4.75
Serum Albumin	Bromocresol Green (BCG)	CRM470	1	0.05	0.132	0.14
			2	0.03	0.132	0.14
Serum Total Proteins	Biuret method	NIST SRM 927c	1	0.07	0.083	0.11
			2	0.05	0.083	0.10
Serum Urate	Uricase, colorimetric	IDMS reference method	1	0.06	0.0642	0.09
			2	0.12	0.0642	0.14
Plasma Glucose	Hexokinase	NIST SRM 965	1	1.8	2.7	3.24
			2	5.64	2.7	6.25
Serum Creatinine	Enzymatic IFCC-IDMS Standardized	NIST SRM 967	1	0.03	0.07	0.08
			2	0.06	0.07	0.09
Serum Urea Nitrogen	Urease, UV	NIST SRM 909b L1	1	0.33	1.62	1.65
			2	0.89	1.62	1.85
Serum Total Bilirubin	DPD	NIST SRM 916a	1	0.03	0.05	0.06
			2	0.06	0.05	0.08
Serum Total Calcium	Arsenazo III	NIST SRM 909b L1	1	0.08	0.08	0.11
			2	0.11	0.08	0.14
Serum Alanine Aminotransferase	UV without P5P	IFCC reference measurement procedure	1	1.1	1.56	1.91
			2	3.03	1.56	3.41
Serum Sodium	ISE indirect	NIST SRM 919b	1	0.75	1.704	1.86
			2	0.79	1.704	1.88
Serum Potassium	ISE indirect	NIST SRM 918c	1	0.02	0.066	0.07
			2	0.05	0.066	0.08
Serum Chloride	ISE indirect	NIST SRM 919b	1	0.63	1.63	1.75
			2	0.64	1.63	1.75
Serum Lactate Dehydrogenase	Lactate to pyruvate	IFCC reference measurement procedure	1	5.43	5.45	7.69
			2	10.93	5.45	12.21
Serum Creatine Kinase	NAC activated	IFCC reference measurement procedure	1	3.86	11.06	11.71
			2	8.51	11.06	13.96
Serum Magnesium	Xylidyl blue	NIST SRM 909b L2	1	0.16	0.02	0.16
			2	0.19	0.02	0.19
Serum Gamma Glutamyltransferase	IFCC	IFCC reference measurement procedure	1	1.11	2.78	2.99
			2	2.12	2.78	3.50
Serum Phosphorus	Phosphomolybdate	Beckman Coulter Master Calibrator	1	0.05	0.03	0.06
			2	0.08	0.03	0.09

**Table 3:** Characteristics of measuring systems and measurement uncertainty contributions for measurands evaluated in Beckman Coulter UniCel DxI 800*. *Beckman Coulter, Inc., Brea, California, United States QC: quality control; U_Rw_: measurement uncertainty for long-term imprecision; U_cal_: measurement uncertainty of the value assigned to an end-use calibrator; U_result_: measurement uncertainty associated with laboratory results

MEASURAND	METHOD	CALIBRATOR TRACEABILITY	QC LEVEL	U_Rw_	U_cal_	U_result_
Serum Triiodothyronine, Free	Chemiluminescence	Manufacturer’s Working Calibrators	1	0.24	0.1	0.26
			2	0.47	0.1	0.48
			3	0.76	0.1	0.77
Serum Thyroxine, Free	Chemiluminescence	Manufacturer’s Working Calibrators	1	0.07	0.039	0.08
			2	0.17	0.039	0.17
			3	0.21	0.039	0.21
Serum Thyroid Stimulating Hormone	Chemiluminescence	Human Thyroid Stimulating Hormone WHO 3rd International Standard 81/565	1	0.02	2.43	2.43
			2	0.19	2.43	2.44
			3	1.63	2.43	2.93

**Table 4:** Characteristics of measuring systems and measurement uncertainty contributions for measurands evaluated in bioMérieux VIDAS*. ^a^ Values higher than the minimum APS are in bold; *bioMérieux SA, Marcy-l'Étoile, France QC: quality control; U_Rw_: measurement uncertainty for long-term imprecision; U_cal_: measurement uncertainty of the value assigned to an end-use calibrator; U_result_: measurement uncertainty associated with laboratory results; IRP: International Reference Preparation

MEASURAND	METHOD	CALIBRATOR TRACEABILITY	QC LEVEL	U_Rw_	U_cal_	U_result_^a^
Serum Luteinising Hormone	Fluorescent Immunoassay	WHO 2nd International Standard 80/552	1	0.1	2.82	2.82
			2	0.8	2.82	2.93
			3	3.16	2.82	4.24
Serum Follicle Stimulating Hormone	Fluorescent Immunoassay	WHO 2nd IRP 78/549	1	0.33	2.02	2.05
			2	1.44	2.02	2.48
			3	2.96	2.02	3.58
Serum Prolactin	Fluorescent Immunoassay	WHO 3rd International Standard 84/500	1	0.66	6.99	7.02
			2	1.29	6.99	7.11
			3	2.39	6.99	7.39
Serum Ferritin	Fluorescent Immunoassay	WHO 1st IRP 80/578	1	4.58	23.2	23.65
			2	11.57	23.2	25.92
			3	30.42	23.2	38.26
Serum Total Prostate Specific Antigen	Fluorescent Immunoassay	WHO 1st IRP 96/670	1	0.04	2.57	2.57
			2	0.16	2.57	2.57
			3	1.23	2.57	2.85
Serum Cancer Antigen 125	Fluorescent Immunoassay	Manufacturer’s Internal standard material	1	0.82	3.55	3.64
			2	3	3.55	4.65
			3	7.85	3.55	8.62

The estimated MU for plasma glucose, serum alkaline phosphatase (ALP), lactate dehydrogenase (LD), creatine kinase (CK), sodium, chloride, and ferritin were higher than the minimum APS limits. It was also observed that u_Rw_ (derived from SD) was small for all the measurands except LD (5.43 and 10.93 for L1 and L2 respectively). It was noted that u_cal_ for ALP (4.7), LD (5.45), CK (11.06), and Ferritin (23.2) were high and this was a major contributor to the high estimated MU for these measurands. Furthermore, the estimated MU for LD Level 2 (10.86) was nearly three times the minimum APS (3.9). We also noted that the minimum APS for sodium (0.40) and chloride (0.74) were exceptionally low. Despite the low u_Rw_ observed in our laboratory, we were unable to achieve these targets.

The analysis showed that most measurands met the minimum APS limits, with a majority also achieving desirable APS, indicating strong analytical performance. However, a few measurands exhibited higher MU, primarily due to elevated calibrator uncertainty. Additionally, some APS targets were exceptionally stringent, making compliance difficult despite low random uncertainty. These findings highlight the impact of calibrator traceability on MU estimates and the need to assess the feasibility of APS for certain parameters.

## Discussion

Metrological concepts such as MU and metrological traceability (MT) are crucial for assessing the accuracy and reliability of clinical laboratory results. These concepts ensure that the measured values provided by the laboratory are precise and that these results remain comparable and transferable across different times and locations. As evident from our study on the Beckman DxC 700 AU analyzer, most of the desirable APS appear attainable.

For serum sodium and serum chloride, the minimum APS were not achieved in our study. Similar findings were reported by Pasqualetti et al., who observed that despite ongoing improvements in electrolyte MU, the results for sodium and chloride still exceeded the APS, whereas potassium met the APS, even at the desirable level, due to its higher biological variability in plasma [15]. Their study demonstrated that MU for electrolytes improved following a targeted manufacturer's intervention on the ISE module fluidics and suggested incorporating this intervention into the routine maintenance of all platforms. Given the high MU for sodium and chloride, we recommend that in vitro diagnostic manufacturers focus on enhancing instrument performance and reducing calibration uncertainty (u_^cal^_), especially when dealing with stringent APS for plasma electrolytes. Similar improvements are necessary for the u_cal_ of ALP at 4.7 and CK at 11.06, which remain considerably high.

In a study by Braga et al., the MU on the Abbott Alinity c system (Abbott Laboratories, Chicago, Illinois, United States) for serum albumin was found to be 3.54 (with the result of approximately 40 g/L), which is approximately twice the minimum APS derived from biological variation, set at 1.88 [5]. In contrast, our study reported an MU for serum albumin of 0.14 at level 1 of IQC, which is well below the desired APS. This significant difference highlights the impact of the analyzer and methodology on MU. Consequently, it underscores the necessity for further studies using different analyzers and methodologies to better understand and standardize MU across various platforms.

We observed that for plasma glucose the estimated MU at IQC level 1 (3.08) slightly exceeded the minimum APS (3.0). Achieving the minimum APS can be accomplished by improving the u_Rw_ (currently 1.49). But, to reach the desirable APS levels, a reduction in the u_cal_ (currently 2.7) is necessary.

Estimating MU offers several benefits. Firstly, it raises awareness among in vitro diagnostic (IVD) manufacturers about the MU budget, potentially influencing the selection of higher-order references. Secondly, it aids in verifying IVD medical device quality. Thirdly, it highlights the results that are clinically unsuitable, thereby encouraging efforts to enhance assay performance quality. Lastly, it facilitates meaningful comparisons between a patient's current value and their previous value of the same type, or with established clinical decision values [16].

MU plays a critical role in clinical decision-making by influencing result interpretation, diagnostic accuracy, and treatment monitoring. High MU can lead to misclassification of patient results, impacting clinical decisions, particularly for analytes with narrow reference ranges or critical thresholds. By including MU in laboratory reports, clinicians gain a clearer understanding of result reliability, allowing them to interpret patient data with greater confidence. This approach helps in reducing diagnostic errors and ensuring appropriate therapeutic interventions. Additionally, laboratories can use MU estimates to identify tests with high variability and communicate these findings to physicians, promoting evidence-based decision-making.

ISO also states that for a result to be applicable in medical decision-making, the MU must be appropriate, and ideally, it should be as low as technically achievable [7]. If some are not achievable, laboratory professionals may recognize the need for industry improvements.

In our study, we utilized biological variation data to determine the allowable MU for most measurands. However, for a more accurate analysis of MU, it is essential to conduct high-quality outcome-based studies. These studies are crucial for deriving APS for measurands that should be assigned to the Milan model [6,17].

Estimating MU and comparing it to APS gives laboratories valuable insights into the variability of their results. This information can be shared with IVD medical device manufacturers to encourage improvements in measurement systems

To meet acceptable MU, it is important to identify all MU contributions in the metrological traceability chain and determine the portion of the total MU budget that could be utilized at each QC level. Braga et al. suggested that the MU of higher-order references should not exceed one-third of the total MU budget, and when combining MU at the commercial calibrator level, no more than half of the budget should be utilized [6]. In our study, we found that improvements in measuring systems are needed to achieve this for measurands like serum CK, LD, ALP, glucose, ferritin, sodium, and chloride. Hence, for these measurands, it is crucial to prioritize the substantial reduction of uncertainty linked to all the levels of the metrological chain.

Further research is required to determine whether other commercial measuring systems can meet these APS or not. Laboratories can enhance clinical decision-making by informing physicians about tests that have high MU. Including MU in laboratory reports encourages clinicians to consider the range of potential outcomes rather than focusing solely on a single numerical value [18]. This approach facilitates more precise and informed clinical diagnoses.

MU should be regarded as a critical quality indicator in clinical laboratories, as it effectively reflects the performance of an IVD medical device as well as the overall quality of the laboratory [10]. Future research should focus on standardizing MU estimation across different analytical platforms and methodologies to enhance result comparability. Large-scale, multi-center studies are needed to establish robust, outcome-based APS for key measurands, particularly those currently relying on biological variation data. Additionally, evaluating the impact of MU on patient outcomes through clinical studies can provide deeper insights into its significance in medical decision-making. Collaborative efforts between clinical laboratories, regulatory bodies, and in vitro diagnostic manufacturers are essential to refine measuring systems, reduce calibration uncertainty, and improve analytical performance. By prioritizing these areas, future research can contribute to more precise and reliable laboratory testing, ultimately enhancing patient care and safety.

One limitation of this study is that it focuses on a single laboratory setting using specific analytical platforms (DxC 700 AU, UniCel DxI 800, and VIDAS), which may limit the generalizability of the findings to other laboratories using different instruments and methodologies. Additionally, while the study estimates MU for 31 measurands, the allowable MU for some analytes was derived from biological variation data rather than outcome-based APS due to the unavailability of relevant literature, which may affect the clinical applicability of the findings.

## Conclusions

Estimating MU offers objective insights into the quality of measurement systems. Its comparison to relevant APS supports the medical applicability of laboratory results. Understanding MU is essential for pinpointing measurands and measuring systems that need analytical improvement; hence, ensuring their reliability for clinical application. Failing to meet APS can lead to misdiagnosis, treatment errors, and patient safety risks. It may also hinder ISO 15189 accreditation and increase retesting costs.
